# Spontaneous Massive Splenic Infarction in the Setting of Renal Transplant and Septic Shock: A Case Report and Review of the Literature

**DOI:** 10.1155/2014/510259

**Published:** 2014-09-15

**Authors:** Christine L. Bokman, Maroun Sfeir, Veer Chahwala, Enrique Ginzburg

**Affiliations:** ^1^DeWitt-Daughtry Family, Department of Surgery, University of Miami Miller School of Medicine and Ryder Trauma Center/Jackson Memorial Hospital, Miami, FL 33136, USA; ^2^Department of Internal Medicine, University of Miami Miller School of Medicine and Jackson Memorial Hospital, Miami, FL 33136, USA

## Abstract

Massive splenic infarction (MSI) is a rare phenomenon that results from compromised blood flow to more than half of the spleen. Causes of MSI include hematological disorders, coagulopathies, infection, and embolization, and, rarely, MSI is spontaneous. The mainstay of treatment is splenectomy. We report the case of a 50-year-old man with a history of renal transplant who presented with diffuse abdominal pain and rapidly developed septic shock. A computed tomographic study (CT scan) of the abdomen demonstrated MSI. The surgical team was consulted for splenectomy but conservative management was maintained and immune function preserved. The patient's clinical condition was resolved over a three-week period. This report demonstrates successful nonoperative management of a spontaneous MSI most likely secondary to hypoperfusion and a hypercoagulable state from both septic shock and renal transplant.

## 1. Introduction

Massive splenic infarction (MSI) is characterized by vessel occlusion, parenchymal ischemia, and subsequent tissue necrosis involving more than half of the spleen. Etiologies of MSI include sickle cell disease and sickle cell variants, coagulopathies, therapeutic embolization of the spleen, sarcoidosis, hematological malignancies, and organ transplant [1–8]. Infection has also been linked to MSI [9, 10]. Spontaneous MSI is an even rarer occurrence. Reports of spontaneous MSI suggest underlying comorbidities as possible causes but do not point towards a definitive source [11, 12]. Splenectomy is typically the choice of treatment, yet splenic preservation is preferred to preserve immune function. We present a case of spontaneous MSI in a man with underlying comorbidities. The patient's kidney transplant, development of infection, and subsequent septic shock serve as possible causes of the MSI by way of a hypercoagulable state and hypoperfusion. He was successfully managed nonoperatively over a three-week period.

## 2. Case Report

**Figure 1:**
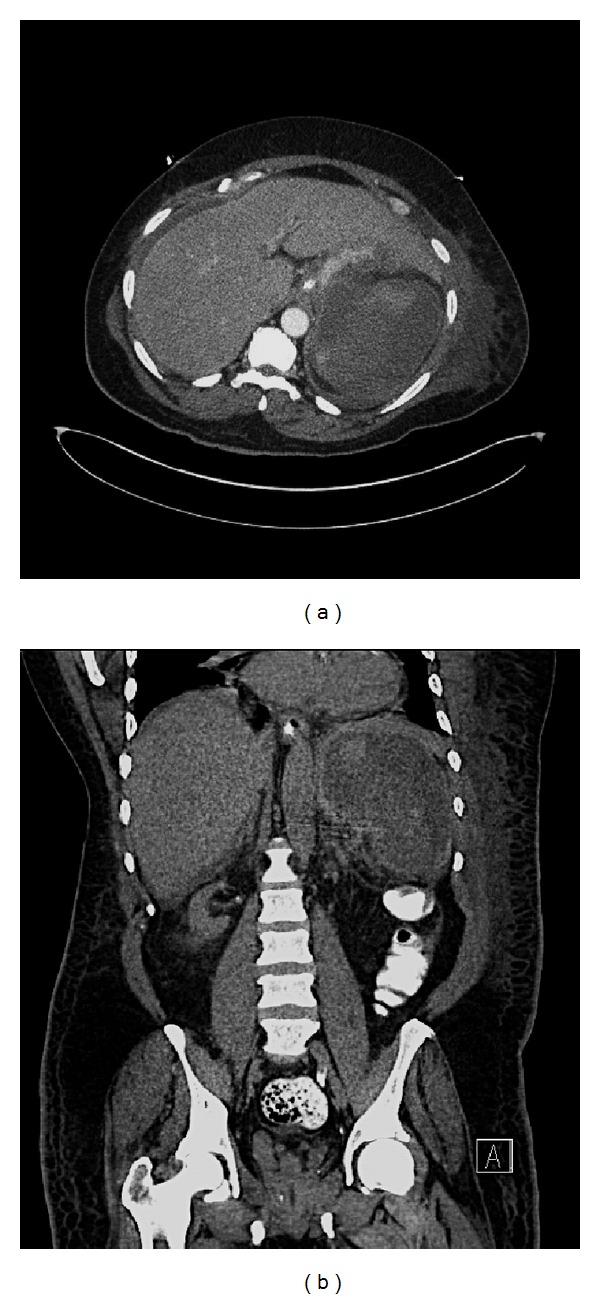
Contrast-enhanced computed tomographic images of the abdomen show massive splenic infarction evidenced by a diffusely hypodense spleen at presentation to emergency department.

A 50-year-old Haitian man was brought by emergency medical services to our emergency department with sudden onset diffuse abdominal pain. The patient had a temperature of 40°C, blood pressure of 180/110, pulse of 120, respiratory rate of 26, and O2 saturation of 98% on room air. His past medical history revealed kidney transplant 18 months earlier due to diabetic nephropathy. Current immunosuppression therapy included tacrolimus and azathioprine. For the past three days, he reported fever and dysuria with no other associated symptoms. Physical examination revealed diffuse abdominal tenderness. An abdominal computed tomography study (CT scan) revealed a diffusely hypodense spleen suggesting massive infarction and perinephric fat stranding of the transplanted kidney consistent with pyelonephritis (Figure 1). Within minutes, the patient became disoriented, hypotensive (80/45 mmHg), and tachypneic (respiratory rate, 40) with labored breathing, requiring intubation. The patient developed acute tubular necrosis with a creatinine of 3.4 mg/dL due to shock. Blood tests showed an elevated white blood cell count (WBC) of 30.7 × 10^9^/L (neutrophils 85.4%) and urine analysis showed 29 white blood cells. The patient was diagnosed with septic shock secondary to pyelonephritis. The patient was evaluated for surgical splenectomy, but medical management was maintained and the patient was admitted to the hospital.

The next day, the patient remained febrile and his renal function continued to deteriorate with a creatinine of 5.6 mg/dL. Urine and blood cultures grew* Enterococcus faecalis* susceptible to ampicillin and vancomycin. Appropriate antibiotic therapy was initiated and a follow-up abdominal CT scan demonstrated stable splenic infarction with no signs of hemorrhage or rupture. Conservative management was maintained to medically treat the infection, and, over the following week, the patient's abdominal tenderness was resolved and blood tests demonstrated a normalizing WBC. The patient was weaned off the ventilator and extubated, which was shortly followed by hospital discharge. Abdominal CT scan eight months later showed a shrunken spleen consistent with an evolving infarct and a functioning transplanted kidney.

## 3. Discussion 

In general, few case reports of MSI have been published [1–12]. The case presented here is of particular significance because it demonstrates the rare occurrence of a spontaneous MSI for which the definitive cause of infarction was unclear. We hypothesize that the MSI most likely resulted from hypoperfusion and a hypercoagulable state due to both septic shock and renal transplant. Well-documented etiologies of MSI are hemoglobinopathies and coagulopathies, which lead to a hypercoagulable state and infarct of the spleen [1–4]. Embolization is also used therapeutically to cause splenic hypoperfusion and can result in MSI [5]. Even infection has been associated with a hypercoagulable state that results in MSI [9, 10].

Bitzer and colleagues report a case of a 27-year-old woman who developed a spontaneous MSI after fulminant meningococcemia [11]. The authors postulated that the cause of the MSI was due to a fulminant reaction to infection and consequent hypercoagulable state. Histological examination of the spleen after splenectomy showed diffuse venous thromboses. Similarly, our patient suffered from septic shock secondary to pyelonephritis, placing him at risk of a hypercoagulable state. The patient was also at risk for hypercoagulability due to prior kidney transplant and current therapy with immunosuppressant drugs. Hypercoagulability is a well-documented complication of renal transplant and immunosuppressant therapy [13, 14]. Additionally, septic shock led to splenic hypoperfusion and further increased the risk of MSI. Capron and coworkers described a cirrhotic patient who developed a spontaneous MSI, which they suggested was due to the increase in oxygen requirement of a congested and enlarged spleen or decreased oxygen-carrying capacity due to anemia [12]. In our patient's case, septic shock led not only to an increase in oxygen demand by tissue but also to an ineffective delivery of oxygen to tissue.

Signs and symptoms of MSI will depend on the cause and extent of infarction. Among the reported cases in the literature, the majority present with left upper quadrant pain [1–8]. The presentation of diffuse abdominal pain in the case of an MSI is unusual and typically occurs if infarction has led to rupture [15]. In our case, however, underlying infection caused the pain to become more diffuse and represented a combination of infarction, graft infection, and septic shock. Prompt imaging also confirmed MSI without rupture. Since the patient demonstrated clinical improvement after being treated with antibiotics, and the MSI was stable on follow-up CT scan, surgical management was avoided and the spleen was preserved.

The role of splenectomy in the setting of MSI remains unclear [1]. Due to the risk of fatal, postsplenectomy sepsis, splenic preservation is preferable [16]. The indications that warrant surgical intervention for any type of splenic infarction are reserved for patients with persistent symptoms, mainly abdominal pain, or the presence of complications, including splenic hemorrhage, abscess, or persistent pseudocyst [17]. Our patient had significant abdominal pain but no other indications for splenectomy even though the spleen was diffusely infarcted. Moreover, splenectomy in the setting of renal transplant has also been associated with an increase in graft rejection [18]. In unique settings such as the one presented, we suggest the choice between medical and surgical management be carefully considered based on clinical signs, physical exam findings, and imaging. Reflex to splenectomy does not always hasten recovery, especially if the cause of MSI is unclear.

## References

[1] Al-Salem AH (2013). Massive splenic infarction in children with sickle cell anemia and the role of splenectomy. *Pediatric Surgery International*.

[2] Abeysekera WYM, de Silva WDD, Pinnaduwa SS, Banagala ASK (2012). Acute massive splenic infarction with splenic vein thrombosis following altitude exposure of a Sri Lankan male with undetected sickle cell trait. *High Altitude Medicine and Biology*.

[3] Beckett D, Miller C, Fernando JR, Banerjee AK (2004). Polycythaemia vera presenting as massive splenic infarction and liquefaction. *British Journal of Radiology*.

[4] Tan DCL, Low AHL, Ong HS, Linn YC, Thumboo J (2006). Unusual abdominal manifestations of catastrophic antiphospholipid syndrome. *The British Journal of Haematology*.

[5] Wholey MH, Chamorro HA, Rao G, Chapman W (1978). Splenic infarction and spontaneous rupture of the spleen after therapeutic embolization. *Cardiovascular Radiology*.

[6] Patel I, Ismajli M, Steuer A (2012). Sarcoidosis presenting as massive splenic infarction. *Case Reports in Rheumatology*.

[7] Aksu T, Erdem AY, Fettah A (2014). Massive splenic infarction and portal vein thrombosis in children with chronic myeloid leukemia. *Journal of Pediatric Hematology/Oncology*.

[8] Troisi R, Hesse UJ, Decruyenaere J (1999). Functional, life-threatening disorders and splenectomy following liver transplantation. *Clinical Transplantation*.

[9] Boivin P, Bernard JF (1990). Pyruvate kinase deficiency, infectious mononucleosis, haemolytic anaemia with cold autoantibodies and massive infarction of the spleen. *Presse Medicale*.

[10] Bonnard P, Guiard-Schmid JB, Develoux M, Rozenbaum W, Pialoux G (2005). Splenic infarction during acute malaria. *Transactions of the Royal Society of Tropical Medicine and Hygiene*.

[11] Bitzer M, Armeanu S, Kröber SM, Horger MS, Erley CM (2003). A young woman with splenic infarction. *The Lancet*.

[12] Capron JP, Chivrac D, Dupas JL, Rémond A, Ossart JL, Lorriaux A (1976). Massive splenic infarction in cirrhosis: report of a case with spontaneous disappearance of hypersplenism. *Gastroenterology*.

[13] Irish AB, Green FR (1997). Environmental and genetic determinants of the hypercoagulable state and cardiovascular disease in renal transplant recipients. *Nephrology Dialysis Transplantation*.

[14] Humar A, Johnson EM, Gillingham KJ (1998). Venous thromboembolic complications after kidney and kidney-pancreas transplantation: a multivariate analysis. *Transplantation*.

[15] Kianmanesh R, Aguirre HI, Enjaume F (2003). Spontaneous splenic rupture: report of three new cases and review of the literature. *Annales de Chirurgie*.

[16] Holdsworth RJ, Irving AD, Cuschieri A (1991). Postsplenectomy sepsis and its mortality rate: actual versus perceived risks. *British Journal of Surgery*.

[17] Nores M, Phillips EH, Morgenstern L, Hiatt JR (1998). The clinical spectrum of splenic infarction. *American Surgeon*.

[18] Sutherland D, Fryd D, So S (1984). Long-term effect of splenectomy versus no splenectomy in renal transplant patients. Reanalysis of a randomized prospective study. *Transplantation*.

